# All-around 3D plant modeling system using multiple images and its composition

**DOI:** 10.1270/jsbbs.21068

**Published:** 2022-02-02

**Authors:** Nobuo Kochi, Atsushi Hayashi, Yota Shinohara, Takanari Tanabata, Kunihiro Kodama, Sachiko Isobe

**Affiliations:** 1 National Agriculture and Food Research Organization, Tokyo 105-0003, Japan; 2 Kazusa DNA Research Institute, Kisarazu, Chiba 292-0818, Japan

**Keywords:** 3D modeling, measurement, multi-view, camera, image, photogrammetry

## Abstract

In this study, we developed an all-around 3D plant modeling system that operates using images and is capable of measuring plants non-destructively without any contact. During the fabrication of this device, we selected a method capable of performing 3D model reconstruction from multiple images. We then developed an improved SfM-MVS (Structure from Motion / Multi-View-Stereo) method that enables 3D reconstruction by simply capturing images with a camera. The resulting image-based method offers a high degree of freedom because the hardware and software can comprise commercially available products, and it permits the use of one or more cameras according to the shape and size of the plant. The advantages of the image-based method are that 3D reconstruction can be conducted at any time as long as the images are already taken, and that the desired locations can be observed, measured, and analyzed from 2D images and a 3D point cloud. The device we developed is capable of 3D measurements and modeling of plants from a few millimeters to 2.4 m of height using this method. This article explains this device, the principles of its composition, and the accuracy of the models obtained from it.

## Introduction

Research and development on an all-around image-based 3D modeling system that is capable of measuring plants non-destructively and without any contact since its seedling stage are conducted in this study. Non-contact measurement methods can be broadly classified into two types: the passive method, which measures 3D coordinates by taking pictures of a subject with a camera from two or more locations and finding the corresponding points of the object in the space, and the active method, which measures those by projecting lasers or patterns of light onto an object, receiving the image with a sensor, and determining the positional relationship between the projected and received light. Both methods are based on the general principle of triangulation. Furthermore, the active methods include those that do not rely on triangulation. For example, the TOF (Time Of Flight) method measures the time that emitted light takes to travel the distance to an object, and calculates its 3D coordinates.

We previously conducted a comparative analysis between these active and passive methods when developing a non-contact, non-destructive all-around plant 3D modeling system (Kochi *et al.* 2021). Based on this analysis, we selected the image-based passive method. We found the active method of using lasers and depth sensors complicated and difficult to handle due to its small degree of freedom. Furthermore, low-cost options are often made of customized products prone to become obsolete. In contrast, the passive method has a high degree of freedom because its hardware and software comprise commercially available products and can be set-up with one or several cameras according to the shape and size of the plant.

Open source software is available for the SfM-MVS method, such as VisualSFM (Wu 2013) and PMVS, (Furukawa and Ponce 2009), and SfM-MVS methods have been widely used in 3D model plant constructions (Liu *et al.* 2017, Lou *et al.* 2014, Ni *et al.* 2016, Pound *et al.* 2014). For taking all-around images to be used for 3D modeling, several imaging capturing systems for the SfM-MVS method have been proposed. For example, Cao *et al.* (2019) and Nguyen *et al.* (2016) proposed an arm for moving a camera around the plants. The systems were considered to be relatively expensive than other systems because the fabrication of the arm that operates a camera required a high level of expertise. On the contrary, Gao *et al.* (2021) and Wu *et al.* (2020) proposed image capturing systems for 3D reconstruction by rotating camera using a turn table. The systems required less cost and specific knowledge for setting an image studio compared to an arm to move a camera, and therefore, have a wider scalability. However, they still required space to move cameras, so it was not suitable in a limited space such as a small studio box.

Meanwhile, Kochi *et al.* (2018) proposed an imaging system for 3D model construction using a turn table for strawberries. In this method, the camera is fixed in one place, and the space required in the studio is smaller than in the system which moves a camera. Therefore, the system reduces the production cost of the image studio. The method proposed by Kochi *et al.* (2018) was able to create a highly accurate model of strawberry with a measurement accuracy within 1 mm. However, in crop breeding, trait evaluation is performed for plants of various sizes and shapes. Therefore, a 3D modeling system that can be applied to various types of plants must be established. In this study, we developed four types 3D modeling systems for plants of height of a few mm to 2.4 m. The developed method was based on the imaging system developed by Kochi *et al.* (2018), and the forward intersection and backward resection mixed methods were applied, which combined the advantages of the forward intersection and backward resection methods (Kochi *et al.* 2021). We expect that the systems will contribute to more precious trait investigations in crop breeding.

## Materials and Methods

### 3D imaging studio

**Fig. 1.**
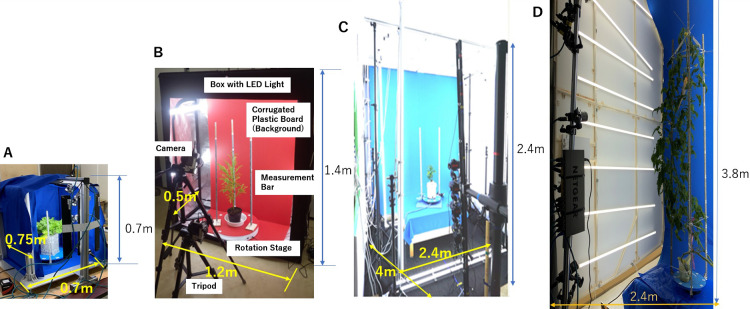
3D imaging device. (A) Small imaging studio corresponding to sizes from few mm to 0.4 m, (B) Low-cost studio corresponding to a size up to 1 m, (C) Mid-size studio corresponding to various plants up to 1.5 m, and (D) Large studio corresponding to a size up to 2.4 m.

We created four types of 3D modeling studios depending on the plant height, ranging from a few millimeters to 2.4 m of height. The studios are shown in Fig. 1. Starting from the left, there is the (A) small 3D studio with four cameras for plants of ~0.4 m of height. This device also corresponds to a growth chamber; (B) compact and low-cost 3D studio using two cameras for specimens of ~1 m; (C) mid-size 3D studio with four cameras for various plants of ~1.5 m of height (Hayashi *et al.* 2019); and (D) large 3D studio using eight cameras for plants up to 2.4 m tall. Studio (A) was put in operation for the analysis of a lettuce (Wada *et al.* 2021), (B) for Japanese cedar (Kurita *et al.* 2020), (C) for soybeans and rice plants, and (D) for plants such as tomatoes, paprika, and eggplants. Table 1 shows size of these studios and specifications of cameras. These devices consisted of the cameras, a turntable, LED lighting, and a measurement bar. The measurement resolutions achieved were approximately 1 mm for studios (A), (B), and (C), and 2 mm for (D).

### Composition of the imaging device

**Fig. 2.**
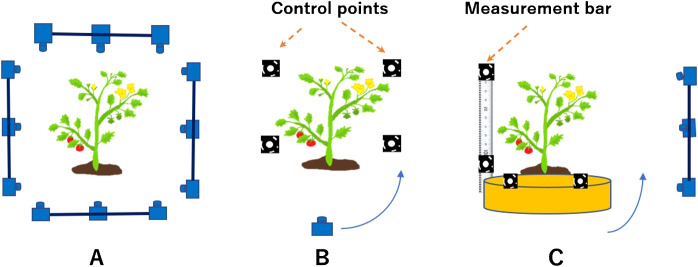
Camera placement using photogrammetry. (A) Forward intersection, which takes images by fixing the cameras, so multiple cameras are needed, (B) Backward resection, which takes images from a single moving camera which requires 3D control points, (C) Forward intersection and backward resection technique, which takes images by moving (rotating) either the object or the camera, it uses a measurement bar, and one or several cameras.

While developing an all-around image-based 3D modeling system, we investigated the advantages and disadvantages of device composition by distinguishing two methods from a photogrammetry perspective (Murai 1989): forward intersection, which fixes the cameras, and backward resection, which takes measurements from a moving camera (Kochi *et al.* 2021). Each method is illustrated in Fig. 2.

The device composition was such that maintenance was not required and 3D model construction was possible with the push of a button; thus, anybody could easily acquire big data for measuring, recognizing, and analyzing using statistics and machine learning. To this end, we developed a forward intersection and backward resection technique that combined the two methods, with the addition of a measurement bar to achieve the intended accuracy.

### Interior and exterior calibration of the cameras

For internally calibrating a camera, its interior orientation (intrinsic) parameters must be determined. These parameters refer to the focal length (principal distance), principal point position, lens distortion, among others, given that accurate and reliable 3D measurements and modeling are not possible if they are not obtained in advance. Normally, autofocus function cannot be used for accurate measurements because it causes the interior orientation (intrinsic) parameters to fluctuate. Therefore, all images must be taken with a fixed focal length provided that the object is in focus. This internal calibration is done using a checkerboard or a similar pattern that is imaged from multiple directions by fixing the focal length of the camera to determine its interior orientation parameters (Noma *et al.* 2002, Zhang 2000). Furthermore, the exterior orientation parameters (extrinsic parameters: i.e., the camera position and attitude) must also be obtained to perform 3D measurements and modeling.

**Fig. 3.**
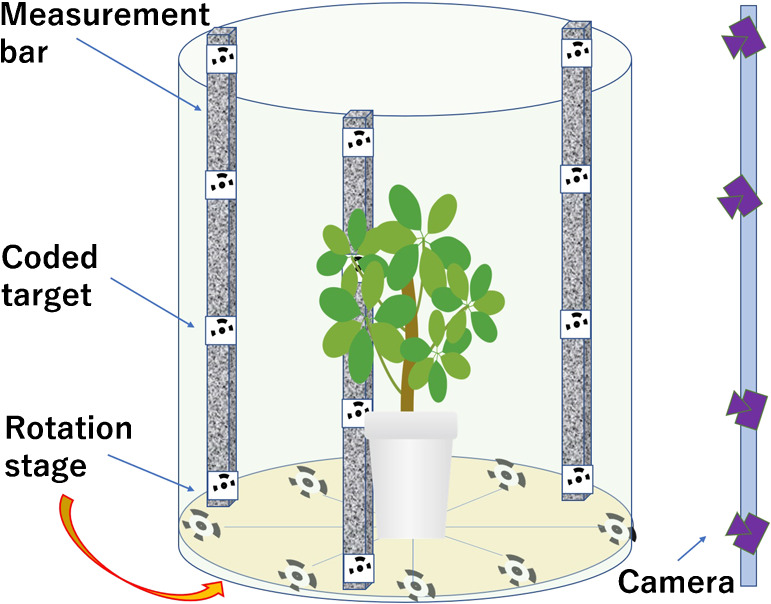
3D imaging device configuration: composed of a measurement bar with random dot pattern, and coded target on a rotation stage. Cameras are arranged in a single vertical row.

However, we eliminated the need for these tasks and established a method that automatically obtains the interior and exterior orientation parameters by calculating and processing them all at once. For this purpose, we devised a measurement bar and developed a method for imaging both the subject plant and the bar simultaneously. A random dot pattern was printed on the measurement bar, and a coded target was also placed on it, as depicted in Fig. 3. The objective was to eliminate the error in the estimation of the camera position and attitude by the addition of characteristic points with a random dot pattern that can be used to securely conducting stereo matching. Another objective was to provide a real scale for the 3D model by automatically detecting and recognizing the coded targets and using the distance between them in the calculations. After set-up, imaging involved capturing images of the object, measurement bar, and turntable simultaneously, all around, by rotating the camera around the plant, or placing the plant on the turntable and rotating it.

For the positioning of this device, the rotation table was rotated in five-degree intervals to create a 360-degree image shot from all angles, for a total of 72 images from each camera. Therefore, all four cameras would take 288 images. This is an adequate number of feature points that can then be used in image processing realized using this method, with the measurement bar shown in each image taken. With this, self-calibrating bundle adjustment (Karara 1989) can be used, making further internal and external calibration no longer necessary.

Self-calibrating bundle adjustment is a method of finding the position of the control points (known 3D coordinates) and common points included in each image within a bundle (light flux) of individual photographs, from which exterior and interior orientation parameters as well as the 3D coordinates of the target space are simultaneously estimated and adjusted using the least-squares method.

Because the interior and exterior parameters can automatically be obtained all at once by conducting the self-calibrating bundle adjustment, the drawbacks of forward intersection—requiring the strict placement of the camera and plant—are no longer an issue; further, an accurate control of the turntable rotation is no longer needed. This reduces the cost of the device and cuts down its maintenance costs.

Furthermore, the disadvantage of backward resection—3D measurement of the control points—is no longer an issue because the coded targets are automatically detected, and the distance between them can be measured. The coded target is divided roughly into two types (Hattori *et al.* 2002): a circular type (Heuvel *et al.* 1992) and a dot distribution type (Ganci and Handley 1998). The color type (Moriyama *et al.* 2010) is also reported, which is put in practical use. Here, we printed and used the circular coded target of Metashape (Agisoft, https://www.agisoft.com/).

The closed-loop method through SfM processing is used for camera position and attitude estimation (Scaramuzza *et al.* 2010); thus, an accurate 3D space can be constructed by connecting and closing the first and last image taken.

### Measurement and processing flow

**Fig. 4.**
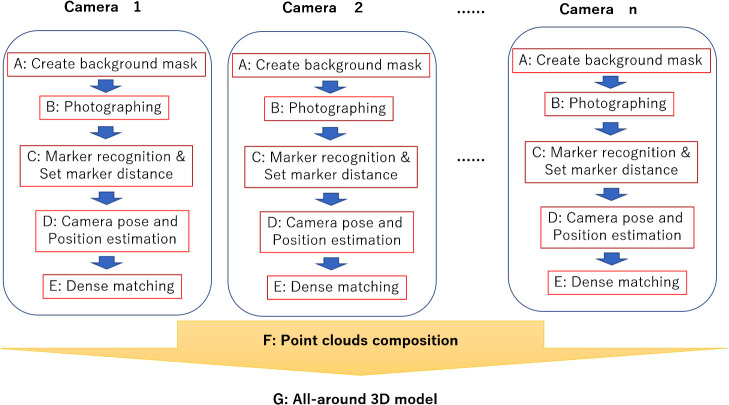
Measurement device composition and measurement and processing flow: automatic processing from (A) to (E) was conducted for each camera. (F) All point clouds were combined, and (G) An all-around 3D model was reconstructed.

Fig. 4 shows the measurement and processing flow chart describing this device. Several cameras were arranged vertically in this system. The method then created a point cloud for each camera, ultimately combining them, making possible its parallel processing. In other words, even after increasing the number of cameras according to the size and shape of the plant, further changes to the algorithm are not required. A background mask was installed and processed for stereo matching to eliminate noise and create a precise 3D model (Hayashi *et al.* 2020).

### Imaging resolution and camera placement

One advantage of this device is that the camera position can be freely arranged according to the shape of the object. This is important because an appropriate camera placement must be set according to the objective at hand for the creation of a precise 3D model.

The measurement resolution at the time of imaging can be expressed as follows with the horizontal resolution *δxy* and depth resolution *δz* when the photographing distance was set as *H*, distance between cameras as *B*, lens focal length as *f*, and the sensor pixel size as *δp* (Matsuoka 2011):

$$\delta xy=\frac{H}{f}\times \delta p$$ (1)

$$\delta z=\frac{H\times H}{f\times B}\times \delta p$$ (2)

**Fig. 5.**
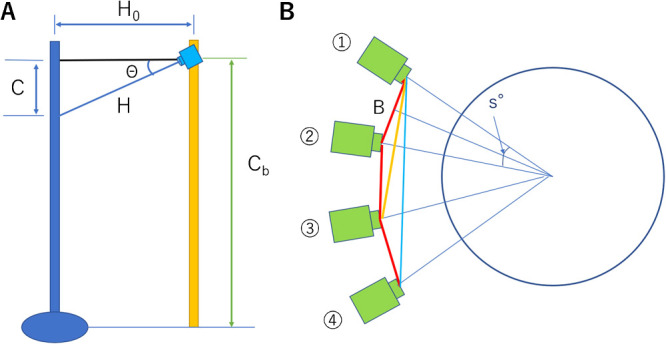
Resolution and camera position. (A) Photographing distance from camera to object is determined by the camera mounting angle *Θ*, (B) Table and camera rotation angle: distance between cameras is determined by rotation angle *s*.

Cases where the camera is inclined, as shown in Fig. 5A, can be expressed by the following equation when setting the horizontal distance from the camera to the object as *H_0_*:

$$c=H_{0}\times \tan\Theta$$ (3)

$$H=H_{0}/\cos\Theta$$ (4)

The relationship between the camera and the plant when it is placed on the rotation table is shown in Fig. 5B, with the camera position rotating from 1 to 4.

If the angle when the camera position rotates from 1 to 2 is set as *s*°, then the distance between cameras *B* from (1) to (2) is as follows:

$$B=2\times H\times \sin\left(\frac{s}{2}\right)$$ (5)

The resolution of the plant, as seen from the camera set at an angle *s*, is determined from equations (3) and (4), when considering the camera installation angle:

$$\delta xy=H_{0}\times \delta p/(f\times \cos\Theta )$$ (6)

$$\delta z=H_{0}\times H_{0}\times \delta p/(2\times f\times H\times \sin\left(\frac{s}{2}\right)\times \cos\Theta )$$ (7)

### Expansion to multiple cameras

When imaging small specimens, for example, a strawberry fruit, measurements can be conducted with this system by the composition of images from a single camera (Kochi *et al.* 2018). For tall plants, they are placed on a rotation table to be imaged and measured using the same processing method by increasing the number of cameras in the vertical direction according to the shape of the plant. When expanding the system in the vertical direction, the angle and installation position need to be determined for each camera according to the plant shape and required resolution.

For this application, we first investigated the arrangement when shooting with four cameras, using soybeans as the imaging subject.

**Fig. 6.**
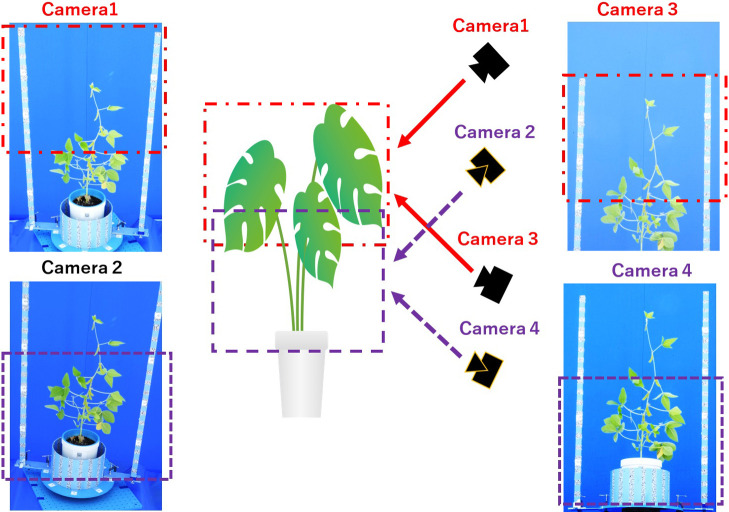
Camera angle and imaging scope: Camera 1 and 2 are set to image from above, and Camera 3 and 4 are set to image from below. Note that pairs of Camera 1 with 3, and 2 with 4 are focused on the same respective areas.

When making measurements of plants with leaves it is often desired to measure the total shape and area of the plants; thus, we want to capture the entire leaf surface without creating visually blocked areas. For example, when imaging a leaf from the side, not only would it be difficult to image the entire leaf but dealing with leaves that are more likely to overlap, creating areas where the leaves cannot be seen. Therefore, we installed a camera arrangement that involves cameras imaging the entire leaf from above and others from below. Fig. 6 describes one of such arrangements. In this case, Cameras 1 and 2 image the plant from above, while Cameras 3 and 4 image the plant from below. Considering that the upper and lower position models are combined during 3D model reconstruction, the imaging ranges of the upper and lower cameras are set to overlap as much as possible. For example, Cameras 1 and 3, and Cameras 2 and 4 were set such that each pair is to image the same range, as shown in Fig. 6. As each point cloud is ultimately combined to produce the entire plant model, these camera arrangements are used to satisfy the required resolution.

## Results

### Actual device settings

The abovementioned points were used as the basic principles for establishing the actual device composition. The resolution was set to be within 1 mm of the target. Table 2 summarizes the camera settings used in a mid-size system corresponding to soybeans up to 1.5 m in Fig. 1C. The camera arrangement was the same as that shown in Fig. 6. A Nikon D5500 digital SLR camera was used (refer to Table 1). The resolution values in Table 2 were calculated by setting the distance from the center of the camera to the center of the turn table *H_0_* as 2.3 m and using equations (6) and (7). The elevation angles of each camera were set to ±25°. Under these conditions, the resolution in the *xy* direction was 0.17 mm, and the depth resolution was 0.63 mm.

Furthermore, Table 3 summarizes the arrangement settings of the eight cameras in the large 3D studio used for a tomato plant capable of doing measurements up to 2.4 m in height as shown in Fig. 1D, as well as the measurement resolution obtained under these conditions. A Lucid Triton TRI1089S-CC camera was used (refer to Table 1). The installation angles of the eight cameras were as follows: the four cameras capturing from above were set to 20°, and the four from below were set to –15°. The distance from the center of the camera to the center of the turntable *H_0_* was 1.52 m. The resolution under these conditions was 0.22 mm for *δxy*; and 0.86 mm and 0.83 mm at 20 and –15 degrees, respectively, for *δz*.

### Camera calibration and measurement accuracy

**Fig. 7.**
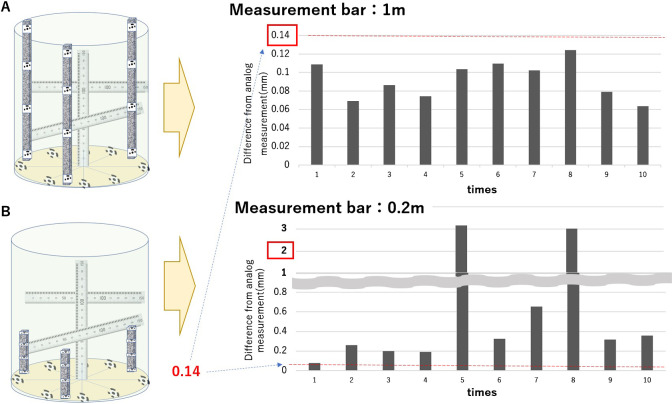
Measurement bar and accuracy: measurement accuracy evaluation of a mid-size studio (Fig. 1C). (A) Measurement bar of 1 m: error was less than 0.14 mm after 10 measurements, (B) Measurement bar of 0.2 m: error was as high as 300 mm.

Fig. 7 describes the method used for measurement accuracy, which involves the use of reference scales (or rulers) installed at the center of the table and at different heights in the vertical and horizontal directions. Then, a 3D model of this assembly was created to verify the length accuracy in each axis. The imaging device that was evaluated was the mid-size studio (Fig. 1c) corresponding to 1.5 m. Measurement accuracy evaluations of this system were conducted by changing the measurement bars height at different times. Two rounds of tests were conducted using measurement bars of 1 m and 0.2 m. The results are shown in Fig. 7 (right). For this evaluation, we marked 14 points in the vertical and horizontal directions, of which 13 points were measured with standard calipers (reading accuracy of 0.01 mm), and the longest section, that was of 0.6 m, was visually measured using a ruler. A total of ten 3D model reconstructions were made and measured. We found that the difference between the actual values and image measurements was less than 0.14 mm in average, and the standard deviation was 0.03 mm in the testing round using a 1-m-measurement bar, when all the points were measured 10 times using calipers (Fig. 7A). Additionally, as shown in Fig. 7B, when range to be measured was not completely covered, because the measurement bars were only 0.2 m tall, the minimum error was over 120 mm, and no meaningful measurements were made. Thus, the measurement bars must be large enough to surround and cover the entire range to be measured.

**Fig. 8.**
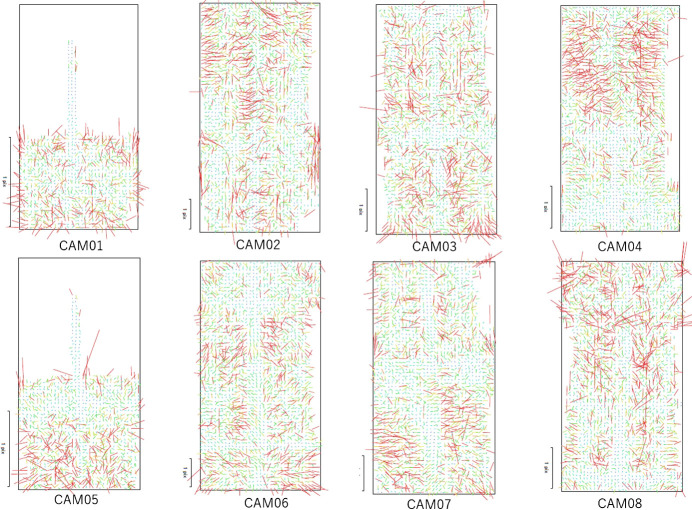
Image residuals by camera calibration: vector residuals of eight cameras in the large studio (Fig. 1D). Sections where the residual vectors are not shown (i.e., white areas) are those where correction was not feasible.

Measurement accuracy evaluations for the large studio corresponding to 2.4 m were conducted following a similar procedure. A total of 30 locations were measured this time, 21 of them showed an average error of 0.95 mm and a standard deviation of 1.24 mm, falling within the 2 mm target. The remaining nine locations performed poorly, having an average error of 4 mm and a standard deviation of 4.4 mm. These poor locations were found at the top and at the edges of the studio. Fig. 8 shows image residuals found at the locations where camera calibration was conducted for each of the eight cameras. CAM01 and CAM05, which imaged the topmost section, could not be corrected because the measurement bar looked short in half of their fields of view. Further, the calibration on both ends of the other cameras was not corrected.

### 3D modeling examples

**Fig. 9.**
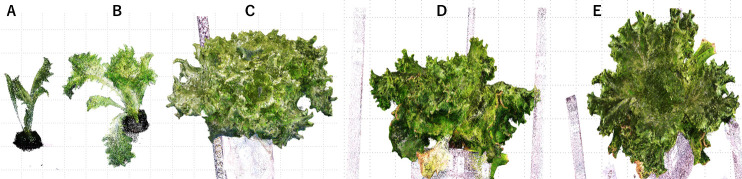
3D model of a lettuce obtained with small studio shown in Fig. 1A. Here (A)–(C) show growth records, (D) Side view image, (E) Top view image.

Fig. 9A–9C show examples of 3D models constructed to record the growth of a lettuce in the small studio (Fig. 1A). Fig. 9D shows its 3D model from the side, and Fig. 9E shows its model from the top. This model was constructed with a resolution of 0.3 mm. As can be seen from the figures, the details of the plant are well expressed, and the shape and even the number of leaves can be counted.

**Fig. 10.**
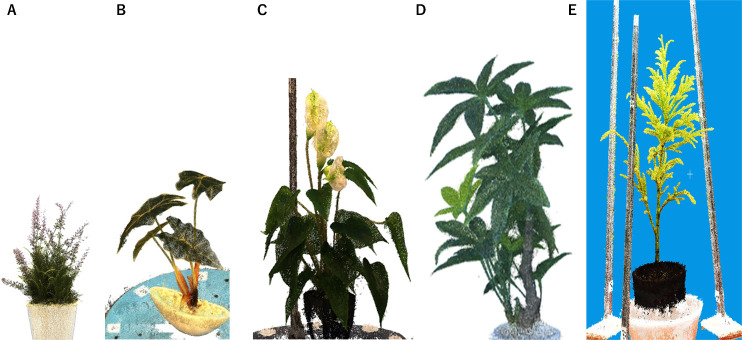
3D models. (A)–(D) 3D models imaged with the 1.5 m mid-size studio shown in Fig. 1C. (E) 3D model of a Japanese cedar using the 1 m studio shown in Fig. 1B.

Fig. 10A–10D show four 3D models created in the 1.5 m studio in Fig. 1C, and Fig. 10E shows one 3D model created in the 1 m compact studio in Fig. 1B.

**Fig. 11.**
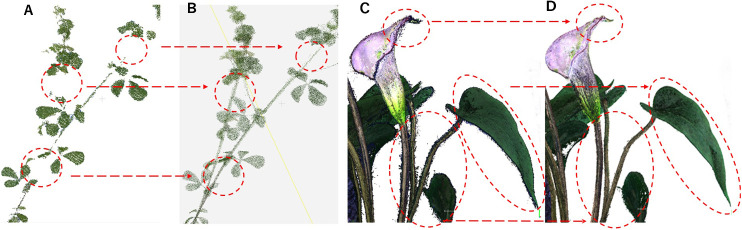
3D models. (A) Metashape, (B) our proposed method, (C) Metashape, (D) our proposed method, the portion enclosed with the circle of figure (A) cannot reconstruct stems, (B) shows reconstructing stems, (C) shows lot of noise with the edge portions of leaves or stems, and (D) shows reduced noise with the edge portions.

3D models reconstructed by Metashape (Agisoft, https://www.agisoft.com/) are shown in Fig. 11A and 11C, and those reconstructed by our proposed method are shown in Fig. 11B and 11D. Fig. 11A shows that the stems are not reconstructed, but Fig. 11B shows that they are. Comparing the noise in Fig. 11C and 11D, Fig. 11C has a lot of noise, but our method (Fig. 11D) reduced noise, and the edge of stems and leaves are clearly reconstructed. These results show that our proposed method is superior.

**Fig. 12.**
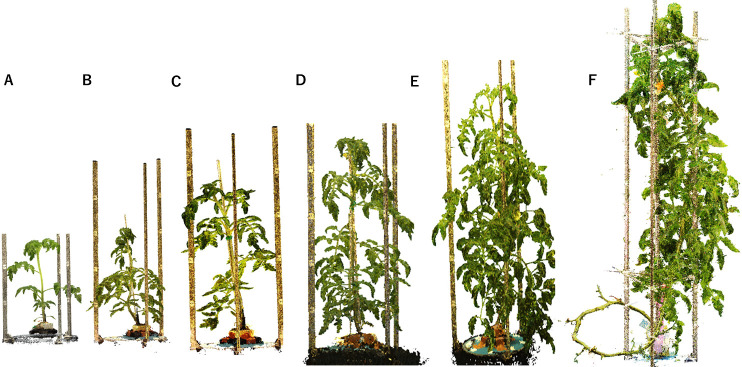
3D models of tomato plant growth records imaged with the large studio shown in Fig. 1D: growth stages. (A) 0.5 m, (B) 0.6 m, (C) 0.8 m, (D) 1 m, (E) 1.5 m, and (F) 2 m (3 m).

Fig. 12 shows the 3D models of a tomato plant having its growth recorded in the large 3D studio corresponding to ~2.4 m in Fig. 1D. The tomato plant grew from 0.5 to 3 m while it was modeled and recorded. A 3D model of a plant of this size has never been created.

## Discussion

Practical 3D measurement methods for plants are mainly divided into active and passive methods. The SfM-MVS method, which belongs to the passive method, is most commonly used in plant 3D modeling. It is furthermore divided into two methods, the forward intersection and the backward resection methods. In the former, camera parameters including camera position and attitude are calculated prior image capturing. In the latter, cameras or objects are moved, and external parameters, such as camera positions and attitudes, are calculated after images are captured from more than three control points. Kochi *et al.* (2021) reviewed these 3D technologies, combined the advantages of the forward intersection and backward resection methods, and proposed the forward-backward mixed calibration method, in which single or multiple cameras were set up in accordance with the complexity of an object, while the subject was being moved. In this study, we developed an all-around 3D plant modeling system by applied the forward-backward mixed calibration method. We constructed four types of 3D imaging studios capable of 3D modeling plants with sizes ranging from a few millimeters to 0.4 m and 2.4 m. We then explained the composition of these devices and their general principles, as well as their measurement resolutions.

Gao *et al.* (2021) and Wu *et al.* (2020) used maize to construct 3D models using the backward resection method, and the measurement errors in comparison to the actual measurement were maximum 2–5 cm (Wu *et al.* 2020) and 1.05 cm (Gao *et al.* 2021). Contrarily, our method had average errors of 0.95 mm at 21 out of 30 measurements and 4 mm at 9 out of 30 measurements, for large plants with a maximum size of 2.4 m. Our system created 3D models with higher accuracy for various sizes of plants than in previously reported methods. We consider that one of the reasons for the high accuracy is the use of the forward-backward mixed calibration method. In addition, we made several minor adjustments, such as photographing the plant while the table was stationary, which was a disadvantage of the turntable method. Furthermore, Gao *et al.* (2021) and Wu *et al.* (2020) used the diameter of a turn table or a pot as a scale to convert the actual measurements, that is, the height direction did not take into account the scale conversion. However, we used the distance between the coded targets on the pillars as the scale, and the three directions (vertical, horizontal, and height) were considered in the conversion.

In this study, we also showed that our system can build 3D models of plants with complex shapes such as lettuce, cedar, and tomato. In crop breeding, various types of traits are evaluated, and the shape is particularly difficult to evaluate using conventional methods. In the conventional method, shape is often investigated by score or using 2D images. However, evaluation using scores are often subjective, and investigation using 2D images results in different values depending on the position of a camera against the subject. However, using a 3D model, it is possible to express the characteristic of shape as a certain quantitative value based on the amount of uneven distribution of point clouds in space (Jiang *et al.* 2019). The device developed in this study is expected to enable more accurate and objective evaluation of shapes.

Another important aspect of plant phenotyping in crop breeding is the ability to measure a large number of individuals. The imaging time using this device is approximately 7–10 min, while the analysis time depends on the subject size and resolution. However, in our experiments, model construction with several tens of millions of points took 1.5 h in the four-cameras arrangement and required 3 h for processing information from eight cameras. From these results, we employed these devices, involving dozens of seedlings being recorded every day, generating the acquisition of large amounts of data, from one or several cameras, in addition to 3D models construction involving batch processing of information running for 24 h. Hence, we considered that our systems are practical for crop breeding where a large number of individuals are evaluated.

The biggest advantage of image-based 3D model construction is that 3D reconstruction can be conducted at any time if the images are already taken, and analyses can be done later with the preferred locations and measurements. In other words, the parts of interest from large amounts of imaging data can be automatically measured and analyzed. This facilitates the implementation of new research, as well as the acquisition and confirmation of new knowledge. Furthermore, this is effective for acquiring and accumulating a large amount of data (big data, e.g.: from hundreds and thousands to millions of data) for machine learning or deep learning. These facts imply that this device will acquire and verify new knowledge in plant research, in large quantities and short durations.

In the future, we intend to (1) disseminate this device, (2) acquire big data for measuring, analyzing, and recognizing using statistics and machine (Deep) learning, (3) conceptualize and create various automatic processing software to acquire new knowledge, and (4) combine image recognition technologies and 3D point cloud processing technologies, establishing new technologies in doing so. We would like to contribute to crop breeding while creating new fields in plant sciences through these efforts.

## Author Contribution Statement

NK and AH conceived of the whole study. YS and KK carried out the evaluation. AH and TT performed image processing. AH and YS conducted the field experiment. NK wrote the manuscript with support from SI. All authors discussed and helped the plant 3D modeling. SI supervised the whole study.

## Figures and Tables

**Table 1.** Size of each studio and specification of cameras

	Small 3D studio (Fig. 1A)	Low-cost 3D studio (Fig. 1B)	Mid-size 3D studio (Fig. 1C)	Large 3D studio (Fig. 1D)
Plant height (m)	~0.4 m	~1.0 m	~1.5 m	~2.4 m
Size (W × D × H: m)	0.7 × 0.75 × 0.7	1.2 × 0.5 × 1.4	2.4 × 4.0 × 2.4	2.5 × 4.0 × 3.8
Camera	Lucid Triton TRI1089S-CC	Sony α6000	Nikon D5500	Lucid Triton TRI1089S-CC
Number of cameras	4	2	4	8
Resolution	4096 × 2160	6000 × 4000	6000 × 4000	4096 × 2160
Pixel pitch (μm)	3.45	3.9	3.91	3.45
Lens (focal length: mm)	8	16	60	25

Lucid: industrial camera, Sony: digital compact mirrorless camera, Nikon: digital SLR camera.

**Table 2.** Mid-size studio (~1.5 m as shown in Fig. 1C) camera settings (four cameras)

	Height (mm)	Angle (°)	Resolution (mm)	
			δxy	δz
CAMERA1	2510	25	0.17	0.63
CAMERA2	2300	25	0.17	0.63
CAMERA3	620	–25	0.17	0.63
CAMERA4	400	–25	0.17	0.63

The camera arrangement was the same as that shown in Fig. 6. The camera measurement resolution was set to be within 1 mm.

**Table 3.** Large studio (~2.4 m as shown in Fig. 1D) camera settings (eight cameras)

	Height (mm)	Angle (°)	Resolution (mm)	
			δxy	δz
CAM01	3310	20	0.22	0.86
CAM02	2680	20	0.22	0.86
CAM03	2050	20	0.22	0.86
CAM04	1590	20	0.22	0.86
CAM05	2040	–15	0.22	0.83
CAM06	1440	–15	0.22	0.83
CAM07	850	–15	0.22	0.83
CAM08	400	–15	0.22	0.83

The four-camera setting described in Fig. 6 was expanded to eight cameras. CAM01–CAM04 represent the cameras set to obtain images from above, and CAM05–CAM08 the cameras set to do it from below. The camera measurement resolution was set to be within 1 mm.
